# Molecular cloning, expression analysis and subcellular localization of four DELLA genes from hybrid poplar

**DOI:** 10.1186/s40064-016-2728-x

**Published:** 2016-07-19

**Authors:** Sian Liu, Lei Xuan, Li-An Xu, Minren Huang, Meng Xu

**Affiliations:** Co-Innovation Center for Sustainable Forestry in Southern China, Nanjing Forestry University, Nanjing, 210037 China; Institute of Botany, Jiangsu Province and Chinese Academy of Sciences, Nanjing, 210014 China

**Keywords:** DELLA proteins, GA signaling, Expression profiles, Protoplast transfection

## Abstract

Gibberellic acid (GA) signaling regulates diverse aspects of plant growth and developmental processes. The DELLA repressors of GA signaling are named for an N-terminal conserved DELLA domain. In this study, four genes encoding DELLA proteins, PeRGA1, PeRGA2, PeGAI1 and PeGAI2, were isolated and characterized in poplar. A gene structural analysis revealed that the DELLA genes were all intron-free. Multiple protein sequence alignments revealed that these proteins contained seven highly conserved domains: the DELLA domain, the TVHYNP domain, leucine heptad repeat I (LHR I), the VHIID domain, leucine heptad repeat II (LHR II), the PFYRE domain, and the SAM domain. Temporal expression patterns of these genes were profiled during the adventitious root development of poplar. The four DELLA genes were expressed in root, stem and leaf in a dynamic manner. The subcellular localization demonstrated that these DELLA genes were mainly localized to the nucleus. These results suggest that the four DELLA genes may play diverse regulatory roles in the adventitious root, stem and leaf development of poplar, and contribute to improving our understanding of conserved and divergent aspects of DELLA proteins that restrain GA signaling in various species.

**Electronic supplementary material:**

The online version of this article (doi:10.1186/s40064-016-2728-x) contains supplementary material, which is available to authorized users.

## Background

Gibberellic acid (GA) signaling plays a pivotal role in plant developmental processes and adaptive responses. The repression by the DELLA repressors can be relieved in response to GA by their rapid GA-induced degradation via the ubiquitin–proteasome system. DELLA proteins, representing a subset of the plant-specific GRAS family of transcription factors, exhibit considerable sequence homology to each other throughout their carboxyl termini, including two leucine heptad repeats(LHRI and LHRII) and three conserved motifs, VHIID, PFYRE and SAW (Davière and Achard 2016). Additionally, DELLA proteins contain an N-terminal conserved DELLA domain, which is involved in recognizing GA signaling and interacts with the GA receptor GIBBERELLIN INSENSITIVE DWARF1 (GID1) (Sun and Gubler 2004; Murase et al. 2008).

Studies have indicated that DELLA proteins negatively regulate the GA signaling pathway and restrain plant growth (Harberd 2003; Achard and Genschik 2009). GA binding to GID1 stimulates the formation of the GA-GID1-DELLA complex, which can induce the degradation of the DELLAs (Davière and Achard 2013). The GA-GID1-DELLA complex can interact with an F-box protein. F-box proteins are components of the SCF (SKP1, CULLIN, F-BOX) E3 ubiquitin-ligase complexes, which catalyze DELLA protein degradation through the 26S proteasome, and then relieve the inhibitory effect of DELLA proteins on plant growth (Lechner et al. 2006). The *Arabidopsis thaliana* genome codes for at least five DELLA proteins that are assigned to three classes (GAI, RGA and RGA-LIKE proteins). RGA and GAI play important roles in cell division and cell expansion in root, hypocotyl, shoot, and floral induction (Dill and Sun 2001; King et al. 2001; Feng et al. 2008; Lucas et al. 2008; Davière et al. 2014). RGL1 modulates floral development (Tyler et al. 2004). RGL2 is a major inhibitor of seed germination (Lee et al. 2002; Cao et al. 2005), and RGL3 contributes to plant fitness during environmental stress (Achard et al. 2008; Wild et al. 2012).

Despite the detailed analysis of GA signaling in several herbaceous model organisms, there are no reports on the isolation and functional characterization of DELLA genes in poplar. In this study, four genes encoding DELLA proteins involved in adventitious rooting were isolated from poplar, and their genomic structures, sequence similarities, expression patterns and subcellular localizations were revealed.

## Methods

### Plant materials

All of the sampled plantlets of the elite clone “Nanlin895” poplar (*Populus deltoids* × *Populus euramericana*) were cultivated on Murashige and Skoog (MS) medium under 16/8 h of light/dark at corresponding temperatures of 25/18 °C. Newly expanded young leaves from 6-week-old plants were used for protoplast isolation and DNA extraction. Various tissues were harvested at multiple developmental stages (1-, 2-, 3-, and 4-week-old roots: 1WR, 2WR, 3WR, and 4WR, respectively, and 4-week-old leaves and stems: 4WL and 4WS, respectively) during adventitious rooting on 4-week-old stem cuttings Additional file 1: Figure S1, then quickly frozen in liquid nitrogen, and then stored at –80 °C until RNA extraction.

### Cloning and sequencing of full-length DELLA genes

Total RNA was extracted from samples using the RNeasy Plant Mini Kit (QIAGEN), and was treated with RNase-free DNase I (TaKaRa). The concentration and integrity of RNA was quantified using the ND-2000 spectrophotometer (Nanodrop) and electrophoresis on 1 % agarose gel respectively. The DNase-treated RNA was used for rapid amplification of cDNA ends (RACE) and then reverse transcribed into cDNA using the PrimeScript RT reagent Kit (TaKaRa) for RT-PCR. Based on probe sequences provided by the GeneChip Poplar Genome Array, nested primers were designed to amplify the full-length sequences with the 3′-Full RACE Core Set Kit and 5′-Full RACE Kit (TaKaRa) according to the manufacturer’s instructions. The PCR products were purified by the QIAquick Gel Extraction Kit (QIAGEN), ligated into pMD19-T vectors (TaKaRa), and then transformed into competent cells of *Escherichia coli* strain TOP10. White colonies were checked by PCR, and the positive colonies were sequenced. By comparing and aligning the sequences of 3′-RACE and 5′-RACE and the middle region products, the full-length cDNA sequences were obtained. The predicted open reading frames (ORFs) were subsequently amplified by PCR, and were verified by sequencing. Genomic DNA was extracted from the newly expanded young leaves using a DNeasy Plant Mini Kit (QIAGEN). Genomic DNA sequences of the above genes were amplified with the RNase-treated DNA, and were verified by sequencing. The sequences of the primers are listed in Table 1.

**Table 1:** Primer sequences of the four poplar DELLA genes

Primer_ID	Forward PCR primer (5′–3′)	Reverse PCR primer (5′–3′)
PeRGA1_3OUTER	CATCAAGAAACCATTGGTGGTGCT	TACCGTCGTTCCACTAGTGATTT
PeRGA1_3INNER	CAAAGCTGAATCTTCTTCTTCGTCAAT	CGCGGATCCTCCACTAGTGATTTCACTATAGG
PeRGA1_5OUTER	CATGGCTACATGCTGACAGCCTA	CCCAACTAGGGTGAGCTTCATTCG
PeRGA1_5INNER	CGCGGATCCACAGCCTACTGATGATCAGTCGATG	ATCGAATTCTTGTTGAGGATAGGCAGC
PeRGA1_ORF	ATGAAGAGAGATCATCAAGA	TCATTGACTCGGTAGCTCGA
PeRGA1_sRT-PCR	AAGCTTGTTTGACTCACTC	ACGTTTCATGTCGCT
PeRGA1_qRT-PCR	GTTGGGTTCAAAGCATG	GGGAATTGCTCTGAGAT
PeRGA2_3OUTER	CACTATAACCCTTCAGATCTCT	TACCGTCGTTCCACTAGTGATTT
PeRGA2_3INNER	TCAACAATCTACCTTCTACTGATCTTGA	CGCGGATCCTCCACTAGTGATTTCACTATAGG
PeRGA2_5OUTER	CATGGCTACATGCTGACAGCCTA	ATTGCATTCCCTGTTTTAAACC
PeRGA2_5INNER	CGCGGATCCACAGCCTACTGATGATCAGTCGATG	CTCGACTTGCATTCGCGAAAGCTTCAAG
PeRGA2_ORF	ATGAAGAGAGATCATCAAGAA	TCATTGTTGTGAATCACCAG
PeRGA2_sRT-PCR	ACTGATCTTGATTCATCTA	TGCCGGCGCAATATTAGACC
PeRGA2_qRT-PCR	CTTAGATTTCCCCAGTAA	CTAGAAAAACCGGACCGT
PeGAI1_3OUTER	CTTACTAGCACCGGTACTATGAC	TACCGTCGTTCCACTAGTGATTT
PeGAI1_3INNER	TGGATGAACTTTTAGCTGTTTTGGGTTA	CGCGGATCCTCCACTAGTGATTTCACTATAGG
PeGAI1_5OUTER	CATGGCTACATGCTGACAGCCTA	ACTCACTGATCCCTCCAACGAG
PeGAI1_5INNER	CGCGGATCCACAGCCTACTGATGATCAGTCGATG	CTCCGGTTTCATTTGTTTCACAACTGAT
PeGAI1_ORF	ATGAAAAGAGAACACTCAA	TTAAGCAGCACCGCCTACTGG
PeGAI1_sRT-PCR	GAGATTGTTACTGTCGTTG	CACTGAGTCAGGGTC
PeGAI1_qRT-PCR	GTCAAACAAATCGGCTT	AATATCTGAGAGAGAGT
PeGAI2_3OUTER	TAAGATCGTCAGACATGGCTGA	TACCGTCGTTCCACTAGTGATTT
PeGAI2_3INNER	CACGCGCAAGAAGATGGTCTTTCCCAC	CGCGGATCCTCCACTAGTGATTTCACTATAGG
PeGAI2_5OUTER	CATGGCTACATGCTGACAGCCTA	CTTCCCCAAGTACACCTCTGAC
PeGAI2_5INNER	CGCGGATCCACAGCCTACTGATGATCAGTCGATG	ATTATGGTTCGCTTCTTGCTCAACAACA
PeGAI2_ORF	ATGAAAAGAGAACACCCA	CTAAGCAGCACCAACTACCG
PeGAI2_sRT-PCR	GCATCTGATTCTGTCCATT	TGGTCGGAGAAATCGATAG
PeGAI2_qRT-PCR	GACCCCTCTGCTGATTCTT	TATAGGTCTGTTTTTAA
EF1α_qRT-PCR	GGCAAGGAGAAGGTACACAT	CAATCACACGCTTGTCAATA
18S_sRT-PCR	TCAACTTTCGATGGTAGGATAGTG	CCGTGTCAGGATTGGGTAATTT

### Bioinformatics analyses

The BioEdit software was used to analyze the DNA and protein sequences. ORFs were predicted by FGENESH program (http://www.mendel.cs.rhul.ac.uk/mendel.php?topic=fgen). The theoretical isoelectric point (pI), molecular weight (MW) and amino acid composition of the proteins were predicted and calculated using Expasy Protparam (http://www.web.expasy.org/protparam/). Protein transmembrane structures, protein domain and signal peptide cleavage site analyses were preformed using the TMHMM, PROSITE and SignalP online tools, respectively. Secondary structures of amino acid sequences were predicted by the SOPMA program (https://www.npsa-prabi.ibcp.fr/cgi-bin/npsa_automat.pl?page=npsa_sopma.html). The phylogenetic tree was constructed using MEGA6 software with the Neighbor-Joining (NJ) method and 1000 bootstraps (Tamura et al. 2013).

### RT-PCR

Total RNA extraction from various tissues and organs, and cDNA reverse transcription were performed as described above. For semi-quantitative RT-PCR, specific primers were designed by Oligo 7 software (Table 1), and they generated a PCR product of 200–400 bp, with the following reaction program: 94 °C for 5 min, followed by 25–28 cycles of 30 s at 94 °C, 30 s at 60 °C and 30 s at 72 °C. For real-time RT-PCR, specific primers were designed to generate an 80–150 bp PCR product (Table 1). Real-time RT-PCR was performed on an ABI ViiA™ 7 Real-time PCR system (Applied Biosystems) using FastStart Universal SYBR Green Master (Rox) for RT-PCR Kit (Roche), according to the manufacturer’s protocol. All reactions were performed in triplicate. The reactions final volume was 20 µL, containing 10 µL of FastStart Universal SYBR Green Master (Rox), 1 µL of each primer, 2 µL of cDNA, and 6 µL dH_2_O. The real-time PCR program was as follows: initial denaturation at 95 °C for 1 min, followed by 40 cycles of 15 s at 95 and 60 °C annealing extension for 1 min. All of the reactions were performed in triplicate. The calculations of relative expression levels between the target and the internal control EF1α (elongation factor 1-alpha) were performed using the delta-Ct method (Xu et al. 2011).

### GFP fusion construct and protoplast transfection

In this study, plasmids were constructed using Gateway technology (Invitrogen), according to the manufacturer’s protocol. The *PeRGA* (or *PeGAI*) coding region (without a stop codon) was cloned into the entry vector, pCR8/GW/TOPO (Invitrogen), by a simple TOPO cloning reaction. For the subcellular localization of tagged proteins, the inset from the entry vector was transferred to its destination vector, p2GWF7, with a C-terminal GFP fusion, using an LR clonase enzyme mix (Invitrogen). The generated GFP fusion vectors (35S::*PeRGA1*-GFP, 35S::*PeRGA2*-GFP, 35S::*PeGAI1*-GFP and 35S::*PeGAI2*-GFP) were high-copy vectors, driven by the promoter of double *35S* cauliflower mosaic virus (CaMV), with ampicillin as the bacterial selection marker. Protoplast isolation and polyethylene glycol-mediated transfection were performed using the method of Tan et al. (2013).

## Results

### Isolation and characterization of DELLA genes

Poplar DELLA genes were successfully isolated and identified by 3′-RACE and 5′-RACE procedures, and termed *PeRGA1*, *PeRGA2*, *PeGAI1* and *PeGAI2*. Comparisons of genomic and cDNA sequences showed that these four DELLA genes were all intron-free. The full-length sequences of *PeRGA1* cDNA was 2321 bp, containing an ORF of 1770 bp, flanked by 324 bp of 5′-untranslated region (UTR) and a 227 bp 3′-UTR; *PeRGA2* was 2504 bp with an ORF of 1824 bp, flanked by 226 and 454 bp 5′- and 3′-UTRs, respectively; *PeGAI1* was 2106 bp with an ORF of 1809 bp, flanked by 144 and 153 bp 5′- and 3′-UTRs; and *PeGAI2* was 2187 bp with an ORF of 1803 bp, flanked by 61 and 323 bp 5′- and 3′-UTRs (Table 2).

**Table 2:** Characteristics of DELLA genes of poplar

Gene_ID	Full-length	5′UTR	3′UTR	ORF	Predicted peptide			Secondary structure prediction			
	cDNA (bp)	(bp)	(bp)	(bp)	MW (kDa)	pI	GRAVY	Hh (%)	Ee (%)	Tt (%)	Cc (%)
*PeRGA1*	2321	324	227	1770	64.15	4.94	−0.240	43.80	16.13	9.00	31.07
*PeRGA2*	2504	226	454	1824	65.88	4.94	−0.193	42.17	13.67	9.72	34.43
*PeGAI1*	2106	144	153	1809	66.31	5.68	−0.279	47.18	15.78	8.64	28.41
*PeGAI2*	2187	61	323	1803	65.96	5.43	−0.242	46.83	15.67	8.17	29.33

*UTR* untranslated region, *MW* molecular weight (kDa), *pI* isoelectric point, *GRAVY* grand average of hydropathicity, *Hh* alpha helix, *Ee* extended strand, *Tt* beta turn, *Cc* random coil

These cDNAs encode polypeptides of 589, 607, 602 and 600 amino acid residues, respectively. The corresponding MWs, pIs and grand averages of hydropathicity (GRAVY) for these polypeptides were 64.15 kDa, 4.94 and −0.240, respectively; 65.88 kDa, 4.94 and −0.193, respectively; 66.31 kDa, 5.68 and −0.279, respectively; and 65.96 kDa and 5.43, −0.242, respectively (Table 2). The SOPMA program was used to predict the secondary structures of these four proteins. The PeRGA1, PeRGA2, PeGAI1 and PeGAI2 proteins contained 43.80, 42.17, 47.18 and 46.83 % of alpha helices, respectively; and correspondingly 16.13, 13.67, 15.78 and 15.67 % of extended strands, respectively; 9.00, 9.72, 8.64 and 8.17 % of beta turns, respectively; and 31.07, 34.43, 28.41 and 29.33 % of random coils, respectively (Table 2).

**Fig. 1:**
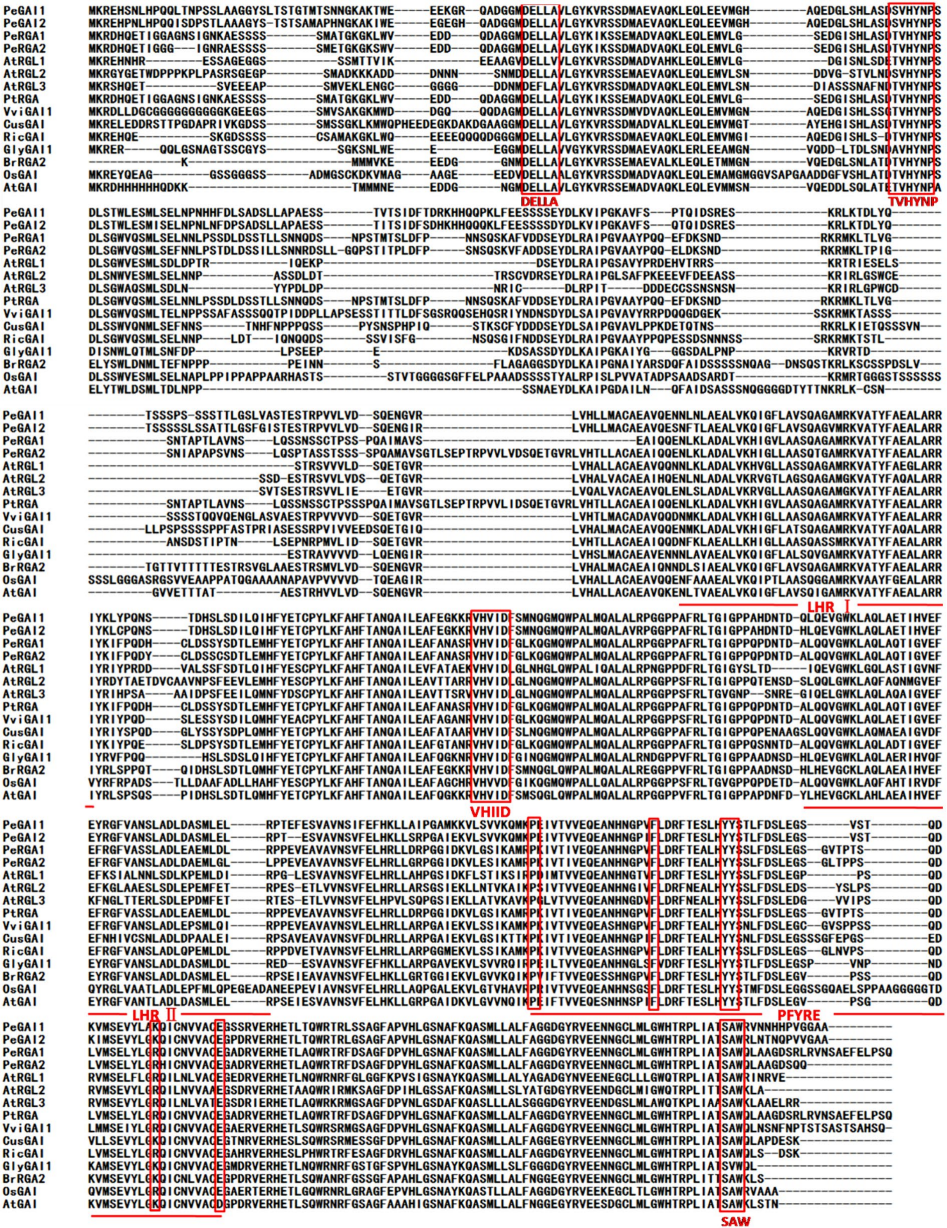
Alignment of the amino acid sequences of various plants DELLA proteins

The multiple alignment of PeRGA1, PeRGA2, PeGAI1 and PeGAI2 with other plant DELLA proteins (PtoGAI, *Paulownia tomentosa*, AFP58844.1; AtGAI, *Arabidopsis thaliana*, Y15193; AtRGL1, *A. thaliana*, AY048749; AtRGL2, *A. thaliana*, NP_186995), AtRGL3, *A. thaliana*, AL391150; PtRGA, *Populus trichocarpa*, XP_002302975.1; VviGAI1, *Vitis vinifera*, XP_002266267.1; CusGAI, *Cucumis sativus*, XP_004155733.1; RicGAI, *Ricinus communis*, XP_002534030.1; GlyGAI1, *Glycine max*, NP_001240948.1; BrRGA2, *Brassica rapavar. Perviridis*, AAX33298.1; OsGAI, *Oryza sativa Japonica*, NP_001051032.1), revealed that the four poplar DELLAs contained two DELLA-specific domains in the N-terminal, DELLA and TVHYNP, and also included five GRAS-specific motifs, LHRI, VHIID, LHRII, PFYRE and SAW, in the C-termini (Fig. 1).

**Fig. 2:**
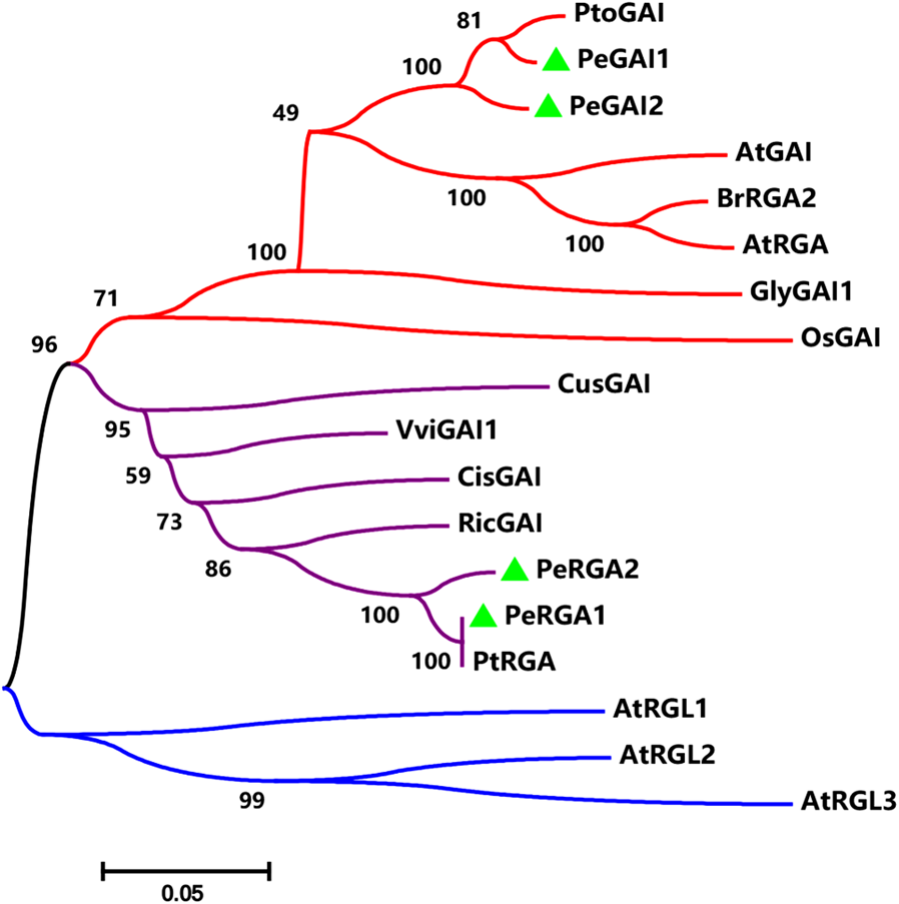
Phylogenetic analysis of the DELLA proteins

To understand the evolutionary relationship of DELLA proteins from different species, the amino acid sequences of 18 DELLA proteins were aligned, and an un-rooted NJ phylogenetic tree was constructed using MEGA 6 software with 1000 bootstrap replications. The un-rooted NJ tree based on multiple sequence alignments showed that the 18 proteins were clustered into three distinct groups (Fig. 2). PeGAI2 and PeGAI2, most closely related to PtoGAI, were positioned in the first clade; PeRGA1 and PeRGA2 most closely related to PtRGA, were positioned in the second clade; and the three RGL proteins of *A. thaliana* were positioned in the third clade.

### Expression patterns of DELLA genes

**Fig. 3:**
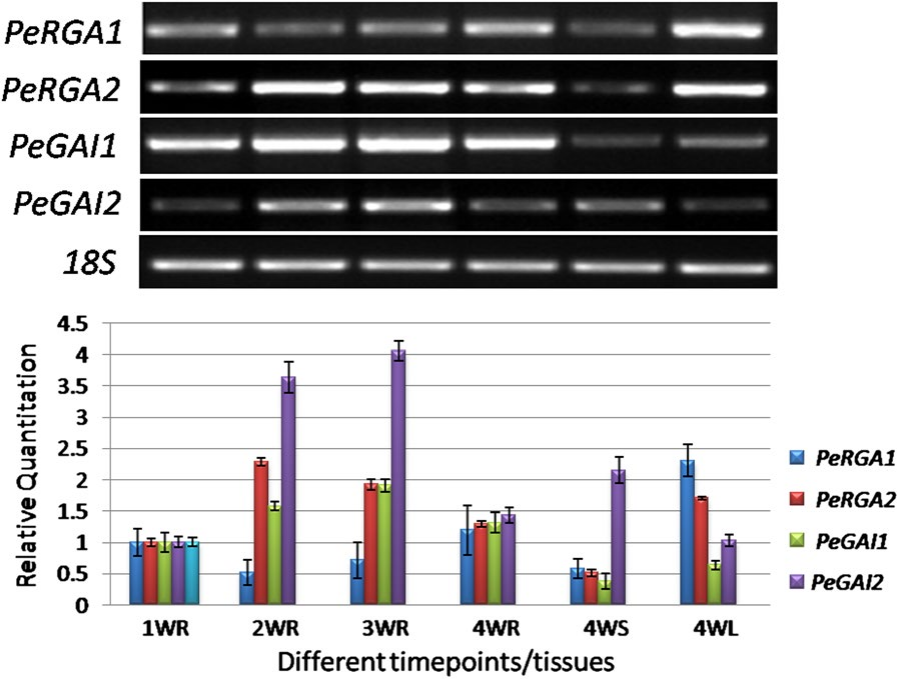
Temporal and spatial expression patterns of poplar DELLA genes by semi-quantitative and real-time RT-PCR. 1WR, 2WR, 3WR and 4WR are 1-, 2-, 3- and 4-week-old roots, respectively; and 4WL and 4WS are 4-week-old leaves and stems, respectively. For semi-quantitative RT-PCR, 18S was used an internal control. For real-time RT-PCR, EF1a was used as an internal control, and the relative transcript levels were calculated using the comparative Ct method. *Error bars* represent standard deviations for three biological replicates

To analyze the expression patterns of the *PeRGA1*, *PeRGA2*, *PeGAI1* and *PeGAI2* genes in poplar, we measured their transcript levels by semi-quantitative and real-time RT-PCR at various developmental time points and in different tissues, including 1WR, 2WR, 3WR, 4WR, 4WS and 4WL Additional file 1: Figure S1. These four *DELLA* genes could be expressed at all developmental time points and in various tissues at different expression levels (Fig. 3). In addition to *PeRGA1*, the mRNA expression patterns of *PeRGA2*, *PeGAI1* and *PeGAI2* were similar at different developmental stages of poplar roots, with an obvious increase–decrease trend during the 1–4 weeks. The minimum of all of them occurred in 1WR, and the maximum in 2WR (*PeRGA2*) and 3WR (*PeGAI1* and *PeGAI2*). However, the expression pattern of *PeRGA1* in contrast with the three genes above, which obviously had the decrease-increase pattern in their 1- to 4-week-old roots, was lower in 2WR and 3WR than in 1WR and 4WR, with a minimum in the 2WR and a maximum in the 4WR. In the root and stem, *PeGAI2* had the highest expression abundance. *PeGAI1* had the lowest expression level in the 4-week-old leaves (Fig. 3).

### Subcellular localization of DELLA proteins

**Fig. 4:**
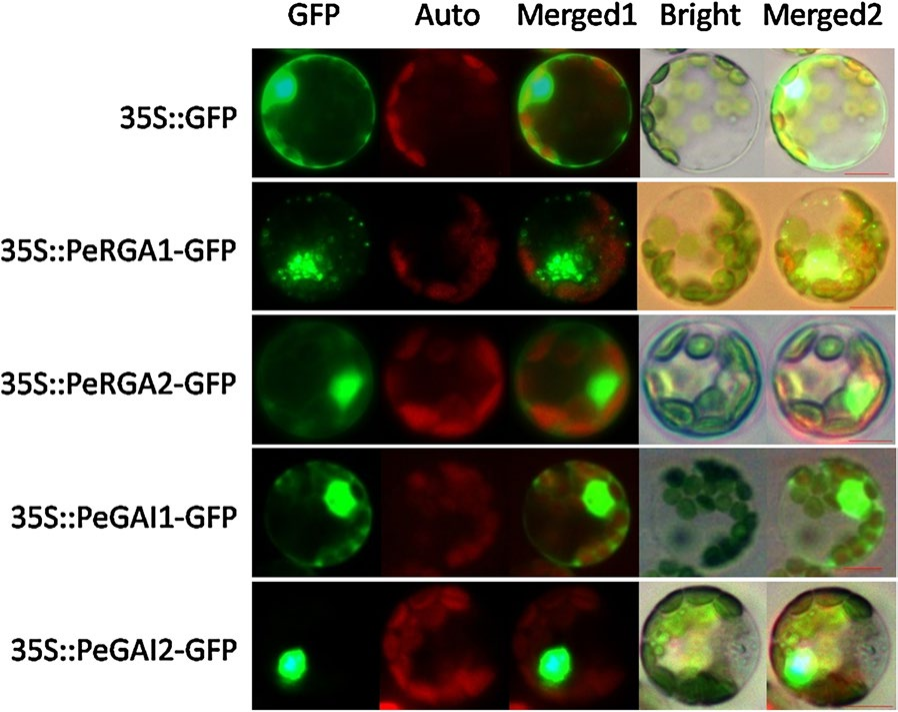
Subcelluar localization of the *Populus* DELLA proteins. Green fluorescence protein (GFP), chlorophyll autofluorescence (Auto), merged 1, bright and merged 2 images are shown. *Scale bar* 5 µm. The 35::GFP fusion was used as a positive protein control, and was detected in the nucleus and cytoplasm in protoplasts

To probe the subcellular localizations of the PeRGA1, PeRGA2, PeGAI1 and PeGAI2 proteins, the GFP-fusion vectors (35S::*PeRGA1*-GFP, 35S::*PeRGA2*-GFP, 35S::*PeGAI1*-GFP and 35S:: *PeGAI2*-GFP) were transformed into *Populus* protoplasts under control of the double *35S CaMV* promoter. Confocal microscopy was used to observe the cellular localization of the fusion proteins. The PeRGA1-GFP and PeGAI1-GFP fusion proteins were located in the nucleus and cytoplasm, while the PeRGA2-GFP and PeGAI2-GFP fusion proteins were located only in the nucleus (Fig. 4). As a positive control, the 35::GFP fusion protein was detected in the nucleus and cytoplasm of *Populus* protoplasts.

## Discussion

DELLA proteins are a subgroup of the plant-specific GRAS family (Bolle 2004). They are highly conserved repressors of GA signaling in angiosperms and their rapid GA-induced degradation can activate the GA signaling pathway (Vandenbussche 2007; Locascio et al. 2013; Zhang et al. 2011). The GA-GID1-DELLA complex plays a pivotal role in controlling plant growth and root elongation (Aleman 2008; Dai 2010; Harberd et al. 2009). The DELLA proteins participation in the growth regulation of roots has also been elucidated, such as, GAI and RGA are the major DELLAs controlling cell expansion in hypocotyl, shoot and root (Fu and Harberd 2003; Ubeda-Tomás et al. 2008). Several lines suggest that active GAs may inhibit lateral root formation (Berova and Zlatev, 2000; Chaney 2003; Watson 2004; Grossi et al. 2005; Gou et al. 2010). By heterologous expression of DELLA-less versions of GAI in *Populus*, the GA signaling was blocked, and the root biomass was increased via lateral root proliferation (Busov et al. 2006). By contrast, GA-overproducing mutations and exogenous GA application in aspen led to suppression of lateral and adventitious root formation (Eriksson et al. 2000).

Our previous study, a whole-genome transcriptional analysis of adventitious rooting in poplar hardwood cuttings was conducted using the GeneChip Poplar Genome Array, revealed the involvement of some putative *DELLA* genes in the adventitious rooting process. Here, four poplar *DELLA* genes involved in adventitious root development were isolated and characterized. A gene structure analysis revealed that these four DELLA genes were all intron-free. Previous studies revealed that *SCR* and *SHR* genes (also belonging to the GRAS family) of *Arabidopsis*, maize, rice and soybean were all intron- free (Xuan et al. 2014), but the homologous genes in *Pinus* contain introns (Laajanen et al. 2007).

Multiple sequence alignments suggest that PeRGA1, PeRGA2, PeGAI1 and PeGAI2 all have DELLA motifs that contain the specific DELLA protein’s domain, and it is necessary for DELLA proteins to interact with GID1 (Sun et al. 2010). The phylogenetic analysis of plant DELLA proteins indicates that these four DELLA proteins of poplar are highly homologous with *Arabidopsis* DELLA proteins. They may have similar functions, being involved in the GA signaling pathway and then regulating poplar root growth (Heo et al. 2011).

Expression patterns of DELLA genes in poplar showed that *PeGAI1* and *PeGAI2* sustained increases in expression levels during adventitious early root formation until root maturity, The *PeRGA2* expression level was highest during middle root development, then began to decline. The expression level of *PeRGA1* was highest in early root development and maturation, but lowest during middle root development. Only *PeRGA1* and *PeRGA2* had high levels of expression in leaves. The three DELLA genes (*PeRGA2*, *PeGAI1*, and *PeGAI2*) have a peak expression in 2 to 3 week-old adventitious root (Additional file 1: Figure S1). This result indicated that the *PeRGA2*, *PeGAI1* and *PeGAI2* might be involved in cell expansion in poplar adventitious root (Fu and Harberd 2003; Ubeda-Tomas et al. 2008). During the growth and development of *A. thaliana*, *RGA* and *RGL2* are the main negative regulators of flower formation and reproductive growth, and *RGA* and *GAI* are the main negative regulators of stem elongation. However, in *A. thaliana*, four DELLA genes are involved in the regulation of seed germination (Peng et al. 1999; Dill et al. 2001; Dill and Sun 2001).

## Conclusions

In this study, four DELLA genes involved in adventitious root development were isolated from poplar, and detailed information about the gene structures, sequence similarities, transcript profile and subcellular localization of the four poplar DELLA genes were revealed. Studies showed that the greater the DELLA protein content in plants, the greater ability to adverse environments (Achard et al. 2006). Therefore, the study of poplar DELLA genes not only increases our understanding of the specific functions of DELLA genes and the DELLA protein pathway in poplar, but also increases our understanding of environmental stress responses in poplar.

## Additional file

10.1186/s40064-016-2728-x Distinct developmental stages of adventitious rooting on poplar stem cuttings.

## Abbreviations

RACE: rapid amplification of cDNA ends
PCR: polymerase chain reaction
RT-PCR: reverse transcription-polymerase chain reaction
ORF: open reading frame
GA: gibberellic acid
